# Chinese Herbal Medicine Paratherapy for Parkinson's Disease: A Meta-Analysis of 19 Randomized Controlled Trials

**DOI:** 10.1155/2012/534861

**Published:** 2012-09-13

**Authors:** Yan Wang, Cheng-Long Xie, Lin Lu, Deng-Lei Fu, Guo-Qing Zheng

**Affiliations:** The Center of Neurology and Rehabilitation, The Second Affiliated Hospital of Wenzhou Medical College, Wenzhou 325027, China

## Abstract

Parkinson's disease (PD) is a common and debilitating neurodegenerative disorder that needs long-term levodopa administration and can result in progressive deterioration of body functions, daily activities and participation. The objective of this meta-analysis evaluates the clinical efficacy and safety of Chinese herbal medicine (CHM) as an adjunct therapy for PD patients. Methodological issues include a systematic literature search between 1950 and April 2011 to identify randomized trials involving CHM adjuvant therapy versus western conventional treatment. The outcome measures assessed were the reduction in scores of Unified Parkinson's Disease Rating Scale (UPDRS) and adverse effects. 19 trials involving 1371 participants were included in the meta-analysis. As compared to western conventional treatment, CHM adjuvant therapy resulted in greater improvement in UPDRS I, II, III, IV scores, and UPDRS I–IV total scores (*P* < 0.001). Adverse effects were reported in 9 studies. The side effects in CHM adjuvant therapy group were generally less than or lighter than the conventional treatment group. In conclusion, CHM adjuvant therapy may potentially alleviate symptoms of PD and generally appeared to be safe and well tolerated by PD patients. However, well-designed, randomized, placebo-controlled clinical trials are still needed due to the generally low methodological quality of the included studies.

## 1. Introduction

Parkinson's disease (PD) is a common, chronic, and progressive neurodegenerative disorder resulting from the death of the dopamine containing cells in substantia nigra and can cause significant disability and decreased quality of life [1]. However, no treatment till now has been shown to be neuroprotective in PD, which can slow down or even halt the progression of the disease [2]. Owing to the absence of disease-modifying therapies, dopamine replacement therapy is still the most effective symptomatic treatment of PD, but this mainstay of pharmacological treatment is eventually complicated by highly disabling fluctuations and dyskinesias [3]. The PD patients continue to experience progressive deterioration of body functions, daily activities, and participation. Thus, near two-thirds of PD patients worldwide resort to various kinds of complementary or alternative medicine, which may possibly influence the motor and/or nonmotor symptoms of PD, and/or the effectiveness of dopaminergic therapy, to alleviate the progressive functional disabilities caused by the disease [4].

 In Mainland China, the prevalence of PD for those aged 65 years or older was 1.7%, which suggested a similar prevalence with the developed countries [5]. However, China faces the largest number of patients with PD because it has one-fifth of the world's population (1.34 billion in 2011). Therefore, the burden of PD prevention and treatment in China is much higher than that in the developed countries. Fortunately, there is one important characteristic of China's national medical system, that is, traditional Chinese medicine (TCM) and western medicine complement and cooperate with each other, being responsible for the health care of Chinese people together [6]. TCM has played an important role in the medical care of PD patients for thousands of years in China [7]. In modern time, TCM therapy is still widely used for PD treatment, and the application covers about three-fourths of the areas in China [6]. In the past decades, several compressive and systematic reviews have focused on TCM for PD treatment [8–10]. However, there is still a lack of reliable scientific evidences for the application of TCM therapy on PD. Recently, some high-quality trials have been published in China [6], and it is timely to reevaluate the existence of evidences. The objective of this meta-analysis therefore is to assess clinical efficacy and safety of Chinese herbal medicine (CHM) as an adjunct therapy of patients suffering from PD.

## 2. Methods

This meta-analysis is conducted according to the preferred reporting items for systematic reviews and meta-analysis: The PRISMA Statement [11].

### 2.1. Eligibility Criteria

Participants were of any age or sex with idiopathic PD diagnosed according to the UK Brain Bank criteria [12] or Chinese National Diagnosis Standard (CNDS) for PD in 1984 [13] or CNDS updated version in 2006 for PD [14]. The CNDS for PD in 1984 [13] is mainly based on clinical observations: (1) to have at least two of the four typical symptoms and signs (bradykinesia, rest tremor, rigidity, and postural reflex disturbance); (2) whether there is atypical symptoms or signs that does not support the diagnosis of idiopathic Parkinson's disease, such as pyramidal signs, apraxia of gait disorders, cerebellar symptoms, intentional tremor, gaze palsy, severe autonomic dysfunction, obvious dementia associated with mild extrapyramidal symptoms; (3) decrease of homovanillic acid in cerebrospinal fluid is helpful for the definite diagnosis of early Parkinson's disease, and for the differential diagnosis of idiopathic tremor, drug-induced parkinsonism, and Parkinson's disease. The CNDS updated version in 2006 for PD [14] was definitions of comparable with the UK Brain Bank criteria [12].

 Interventions were any form of CHMs in any dose as adjunct therapy for PD. The patients at the trial groups were given CHM therapy in addition to western conventional medication (WCM).

The outcome measures included the evaluation with Unified Parkinson's Disease Rating Scale (UPDRS) [15], and the adverse events at the end of the treatment course lasting for at least 12 weeks (3 months). The UPDRS has long been used as the major rating scale that is used for assessing severity of symptoms of PD. The UPDRS scale consists of the following four segments: Part I (mentation, behavior, and mood) addresses mental dysfunction and mood; Part II (activities of daily living, ADL) assesses motor disability; Part III (motor section) evaluates motor impairment; Part IV (complications) assesses treatment related motor and nonmotor complications.

Only randomized controlled trials (RCTs) were included in the study, regardless of blinding, publication status or language. Quasi-RCTs were not considered such as using the admission sequence for treatment allocation.

### 2.2. Search Strategy

We electronically searched CENTRAL (The Cochrane Library 2011, Issue 1), PubMed (1950–April 2011), EMBASE (1980–2010), China Hospital Knowledge Database (CHKD, 1979–April 2011), and Wanfang Med Online Database (WMOD, 1998–April 2011). A list of Chinese and English journals that had the potential to include eligible studies was hand-searched. A manual search of conference proceedings relevant to this topic, references from relevant reports of clinical trials or review articles was performed to retrieve all potentially relevant published and unreported material.

The following search strategy was used: the cross-referenced TCM/CHM and its proprietary names with PD and its derivations, all as MeSH and as free-text words. The Medical Subject Headings (MeSHs) and text keywords TCM/all subheadings, CHM/all subheadings in combination with Parkinson's, Parkinson's disease, and PD were utilized.

### 2.3. Study Selection and Data Extraction

Two review authors (WY, XCL) independently scanned the titles and abstracts to select potential references. Full articles for all potentially relevant trials were retrieved. The two review authors then independently read the selected papers and made a final selection decision. All disagreements were resolved by discussion or by involving a third party author (ZGQ).

A standardized data extraction form was used to extract data, including patients, methods, interventions, and outcomes. The reasons for the exclusion of studies were recorded accordingly. For eligible studies, two review authors (WY, XCL) extracted the data independently. Disagreements were resolved through consultation with a third party author (ZGQ).

### 2.4. Risk of Bias in Individual Studies

Two review authors (WY, XCL) independently assessed the risk of bias of included studies, using the twelve criteria recommended by the Cochrane Back Review Group [16]. The items were scored with “yes (+),” “no (−),” or “unsure (?).” Studies were categorized as having a “low risk of bias” when at least six of the 12 criteria were met. We resolved any disagreement through discussion or consultation with a third party author (ZGQ).

### 2.5. Data Synthesis and Analysis

We analyzed the data using Review Manager (version 5.0). A fixed-effects model or random-effects model was used to investigate the effect of CHMs on PD across the trials, and weighted mean difference was calculated. Heterogeneity between trial results was tested using a standard chi-square test and we also calculated the *I* ^2^ statistic. Funnel plot analysis is used to detect Publication Bias. The two-tailed *P* values less than 0.05 were considered statistically significant.

## 3. Results

### 3.1. Description of Studies

**Figure 1:**
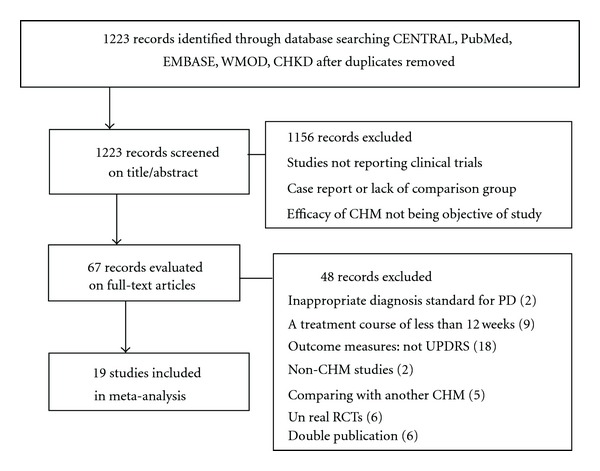
Flow diagram for the process of identifying eligible randomized controlled trials. WMOD: Wanfang Med Online Database; CHKD: China Hospital Knowledge Database; CHM: Chinese herbal medicine; PD: Parkinson's disease; UPDRS: the Unified Parkinson's Disease Rating Scale; RCT: randomized controlled trial.

We identified 1223 potentially relevant articles. After screening titles and abstracts, 1156 were excluded because they were studies with nonclinical trials, case reports, lack of comparison group, or efficacy of CHM not being the objective of study. We conducted full-text evaluation on the remaining 67 articles, and 48 more articles were excluded for not meeting our inclusion criteria: 2 articles used expert-made diagnosis standard for PD; 9 articles reported a treatment course of less than 12 weeks; 18 articles used homemade rating systems or the Webster rating scale, not UPDRS; 2 articles did not study CHM; 5 articles evaluated CHMs paratherapy by comparing combination treatment of CHM and WCM, or another CHM; 6 studies were not real RCTs; 6 studies were suspected of being published more than once by the authors or publishers. Finally, 19 trials were included in this analysis [17–35]. The screening process is summarized in a flow diagram (Figure 1).

### 3.2. Characteristics of Included Studies

A total of 1371 participants were included in the 19 studies. All of the trials were conducted in China. 2 articles published in English [27, 35] and 17 articles in Chinese from 2003 to 2011. 16 studies were single-center trials, while the remaining 3 were multicenter trials [26, 28, 29]. There were 825 male and 546 female participants ranging from 35 to 81 years old. 12 studies applied the CNDS (1984 version) for PD; the other 7 studies used CNDS (updated version in 2006) [30, 31, 34] or UK Brain Bank diagnostic criteria [27, 28, 33, 35] for PD. The disease duration ranged from 6 months to 21 years. Except 7 trials [18, 19, 23, 24, 30–32], the Hoehn & Yahr (H & Y) stage was conducted in 12 trials. All oral CHMs interventions as add-on therapy were investigated by comparing with WCM controls. 4 trials have WCM plus placebo control [26–28, 35]. The course of treatment in all included trials lasted at least 12 weeks (3 months). The details of the characteristics of included studies are listed in Table 1.

### 3.3. Risk of Bias in Included Studies

The twelve criteria recommended by the Cochrane Back Review Group were used to assess the risk of bias [16]. The number of criteria met varied from 2/12 to 11/12 (see Table 2). All the included studies indicated randomization, but only 8 trials reported the method of generating random sequences [17, 18, 26–29, 34, 35], and 5 trials described allocation concealment [18, 26, 28, 29, 35]. 5 trials mentioned blinding procedures to both patients and investigators [26–29, 35], but only one trial was assessor-blind [26]. 3 trials described intention-to-treat analysis [26–28]. 2 trials reported data on dropouts [27, 28]. With exception of 1 trial [26], selective reporting was found in almost all of the trials. Baseline similarity was described in all the studies, but 7 trials did not mention the H & Y stage [18, 19, 23, 24, 30–32]. 15 trials reported constant cointervention, whereas 4 studies were ambiguous [18, 25, 30, 31]. All of the included studies appeared to have acceptable adequate compliance and similar timing outcome assessments. In general, 14 RCTs were deemed to have an unclear risk of bias based on the Cochrane Risk of Bias tool, and the remaining 5 trials are high-quality clinical trials [26–29, 35].

## 4. Synthesis of Results

### 4.1. High-Frequency Herbs Found in TCM Prescriptions for PD

 Based on our review, we documented and ranked the top 16 individual Chinese herbs for PD treatment that were used more than 3 times in the TCM prescriptions of the 19 included trials (Table 3). For example, Prepared Rehmannia Root, White peony Alba, Szechwan Lovage Rhizome, and Tall Gastrodis Tuber are the top 4 most frequently used herbs. The main effects of these herbs include replenishing blood and tonifying Yin, calming the liver, checking endogenous wind, dispelling evil-wind, and activating blood flow. These high-frequency herbs may contribute in composing a fundamental prescription for clinical PD treatment and seems worthy of additional, indepth study.

### 4.2. UPDRS I Scores

**Figure 2:**
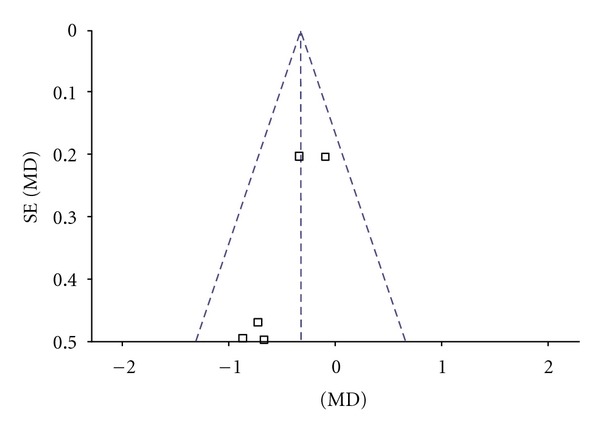
Funnel plot of comparison: Chinese herbal medicine versus conventional treatment: UPDRS I scores.

The 5 independent trials showed the homogeneity in the consistency of the trial results, chi-square = 3.69 (*P* = 0.45); *I* ^2^ = 0%. Thus, fixed-effects model should be used for statistical analysis. Compared to conventional treatment, CHM paratherapy significantly improved UPDRS I scores (WMD −0.33, 95% CI −0.58 to −0.08; *Z* = 2.60 (*P* < 0.001)). The difference suggested that CHM paratherapy was more effective than conventional treatment for symptoms of mentation, behavior, and mood in patients with PD (Table 4). The funnel plot was roughly symmetric. There would be little publication bias for the 5 independent trials (Figure 2).

### 4.3. UPDRS II Scores

**Figure 3:**
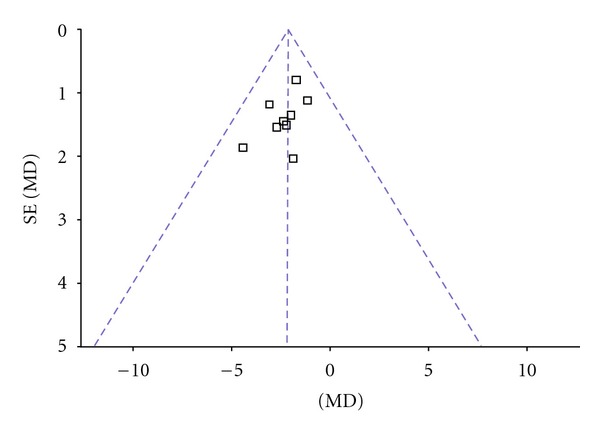
Funnel plot of comparison: Chinese herbal medicine versus conventional treatment: UPDRS II scores.

The 9 independent literatures showed homogeneity in the results of trials, chi-square = 3.26 (*P* = 0.92); *I* ^2^ = 0%. Thus, fixed-effects model should be used for statistical analysis. Compared to conventional treatment, CHM paratherapy significantly improved UPDRS II scores (WMD −2.18, 95% CI −3.03 to −1.33; *Z* = 5.03 (*P* < 0.001)), suggesting that CHM paratherapy could contribute to improving the activities of daily life (ADLs) in patients with PD (Table 5). The funnel plot was symmetric. No evidence of publication bias was found (Figure 3). 

### 4.4. UPDRS III Scores

**Figure 4:**
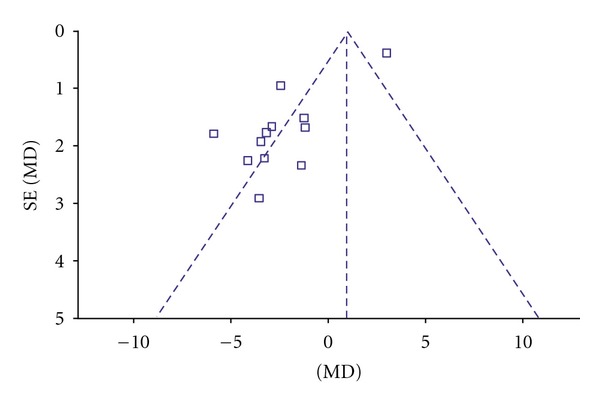
Funnel plot of comparison: Chinese herbal medicine versus conventional treatment: UPDRS III scores.

The 12 independent trials did not show homogeneity in the trial results, chi-square = 89.22, (*P* < 0.001); *I* ^2^ = 88%. Thus, random-effects model should be used for statistical analysis. Compared to conventional treatment, CHM paratherapy significantly improved UPDRS III scores (WMD −2.35, 95% CI −4.61 to −0.08; *Z* = 2.03 (*P* < 0.05)). This result suggested that CHM paratherapy could contribute to improving motor function in patients with PD (Table 6). The funnel plot was markedly asymmetric. There exists a publication bias in the 12 independent trials (Figure 4).

### 4.5. UPDRS IV Scores

**Figure 5:**
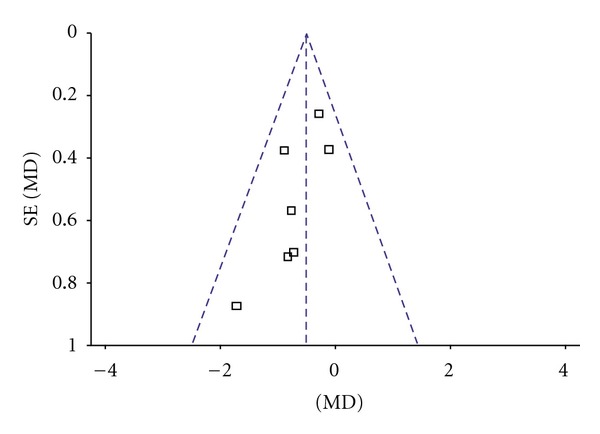
Funnel plot of comparison: Chinese herbal medicine versus conventional treatment: UPDRS IV scores.

The 7 independent studies showed homogeneity in the trial results, chi-square = 5.21 (*P* = 0.52); *I* ^2^ = 0%. Thus, fixed-effects model should be used for statistical analysis. Compared to conventional treatment, CHM paratherapy significantly improved UPDRS IV scores (WMD −0.51, 95% CI −0.83 to −0.20; *Z* = 3.61 (*P* < 0.05)), suggesting that CHM paratherapy could contribute to improving complications of treatment in patients with PD (Table 7). The funnel plot was obviously asymmetric. There exists a publication bias in the 7 independent trials with mainly positive results (Figure 5).

### 4.6. UPDRS I–IV Total Summed Score

**Figure 6:**
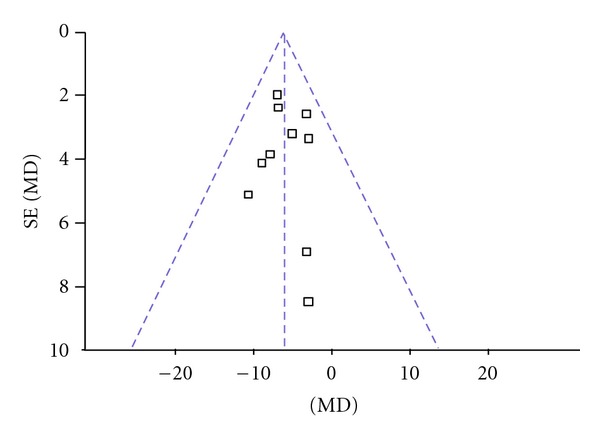
Funnel plot of comparison: Chinese herbal medicine versus conventional treatment: UPDRS I–IV total scores.

The 10 independent trials showed homogeneity in the trial results, chi-square = 4.25 (*P* = 0.89); *I* ^2^ = 0%. Thus, fixed-effects model should be used for statistical analysis. Compared to conventional treatment, CHM paratherapy significantly improved UPDRS I–IV total summed score (WMD −6.09, 95% CI −8.08 to −4.10; *Z* = 6.00 (*P* < 0.001)), suggesting that CHM paratherapy could contribute to improving symptoms of PD (Table 8). The funnel plot showed nearly complete symmetry. No publication bias was found in the 10 independent trials included (Figure 6).

### 4.7. Adverse Effects

Adverse effects were reported in 10 studies [17, 20, 22–24, 27–29, 32, 35], but no mention of side effects in the other 9 trials was reported (Table 9). There were no significant differences in the results of blood routine, urine routine, liver function, renal function, or electrocardiograph (ECG) in both groups of patients before and after treatment [20, 22, 28]. Diarrhea [27, 28], constipation [20, 23, 29], nausea and/or vomiting [17, 20, 23, 24, 29, 32], dry mouth [17, 20], and dizziness [17, 23, 24] were reported in CHM paratherapy group. Other adverse effects including arrhythmia [24], epigastric pain [29], sialorrhea, hypotension, insomnia, and depression [32] were reported. However, no life-threatening adverse effects were noted in these studies, and the side effects were less than or lighter than the conventional treatment group.

## 5. Discussion

### 5.1. Summary of Evidence

The main findings of this meta-analysis were that CHM adjuvant therapy could improve the clinical symptom severity scores for PD and has few adverse effects in comparison to WCM controls. The evidences of CHM paratherapy for PD are emerging and the evidences presented in this meta-analysis potentially benefit a clinical recommendation in spite of some methodological weaknesses. However, there was still not enough replicable evidence to conclude that any specific CHM therapy is effective for WD.

The CHMs evaluated in this paper generally appeared to be safe and well tolerated in patients with PD. However, the safety for the use of CHMs could not be confirmed because only 47.37% (9/19) studies mentioned the safety of interventions or investigated adverse effects. It is recommended that more attention should be given to both recording and reporting the adverse effects of these interventions.

### 5.2. Limitations

There are a number of inherent and methodological limitations to this meta-analysis. First of all, none of included studies had been registered. In September 2004, the members of the International Committee of Medical Journal Editors (ICMJE) published a statement requiring that all clinical trials must be registered in order to be considered for publication [36]. Clinical trial registration will improve research transparency and will ultimately strengthen the validity and value of the scientific evidence base. Thus, the inherent limitation of this paper existed in the primary studies.

 One of the major limitations was the application of various kinds of CHMs add-on therapy used in different trials. They differ in composition, dosage preparation, and methods and manufacturing standards. It is difficult to assess the effect of a particular CHM by means of the evidence synthesis of studies.

There are many methodological weaknesses in this meta-analysis. (1) Randomization: all included studies claimed randomization. However, only 8/19 trials provided sufficient information on how the random allocation was generated such as from random-number table, calculator or computer random-number generator; 5/19 trials reported allocation concealment such as sealed envelopes or a telephone call to the research centre. The proper randomization in RCTs is necessary to avoid selection bias and confounding. Thus, an invalid method of randomization could have distorted our results. (2) Blinding: with exception of blinding (participants and care providers) in 4 trials, the other 15 studies were lack of any blinding method which can produce performance bias and detection bias. Blinding of the outcome assessor was only used in one study. Thus, assessment of outcomes was prone to significant systemic errors. (3) Analysis of data from RCTs: dropouts were only reported in 3 trials, and 1 trial of intention-to-treat analysis was mentioned. Therefore, the results generated from these studies should be interpreted with caution. (4) Placebo controlled: only 4 trials out of the 19 included studies have placebo control. The other 15 trials used an “A + B versus B” design where patients were randomized to receive a CMH paratherapy plus WCM control treatment versus WCM control treatment without a rigorous control for placebo effect. Thus, the results of these studies would be positive because of nonspecific placebo effects [37]. (5) Sample size: the included studies were of relatively small sample sizes in individual trials and without formal sample size calculation. Trials that lacked proper sample size estimation placed their statistical analysis's validity in doubt. The results were likely to be underpowered. (6) Heterogeneity: the imbalance in gender, ethnicity, and wide range in disease duration further compromised the validity of the included trials. Furthermore, outcome measures used in the trials were heterogeneous and incomplete. Thus, the results might have been compromised by the heterogeneity within each CHM intervention and by the study design.

Another limitation was publication bias. Publication bias was assessed by visual inspection of funnel plots. There was bias with UPDRS III and IV score in CHM paratherapy plus conventional treatment compared to conventional treatment alone. In a total of 19 studies, results were all positive in CHM paratherapy group. Therefore, the validity of inferences that can be drawn is threatened.

## 6. Conclusions

### 6.1. Implications for Practice

This is the first meta-analysis of randomized, controlled trials to assess the efficacy and safety of CHM paratherapy in patients with PD. In our meta-analysis, patients receiving CHM adjunct therapy plus WCM exhibit significant improvement in their PD symptoms as evidenced by improvements in their UPDRS scores compared to WCM controls in spite of some methodological limitations. According to the safety assessment of this meta-analysis, the CHM add-on therapy for PD is generally safe and well tolerated. Therefore, CHM paratherapy may be effective and well tolerated for the symptomatic treatment of PD. However, various kinds of CHMs paratherapy were used in different trials. As such, treatment choices must be consider each individual's CHM. Although acknowledging the limitations of this meta-analysis, our findings present several high-quality trials [26–29, 35] and provide potential evidences that CHM adjunct therapy can additionally benefit relieve symptoms of PD. However, methodological robust trials are still needed to further evaluate this therapy due to the generally low methodological quality of the included studies.

### 6.2. Implications for Research

A number of implications for research arise from this paper. First, improvement in the methodological quality of randomized controlled trials is critical for later trials and more methodologically rigorous studies are needed in this field. Second, the included trials were generally of small sample size. None of the trials reported the method of sample size determination. Sample size calculation should be conducted before enrollment. Third, two ways are performed globally for clinical trial transparency: (1) all clinical trials must be registered before the enrollment of the first patient, based on ICMJE statement; (2) the making and dissemination and implementation of reporting standards of clinical trial represented by CONSORT [38] series. In China, CONSORT for TCM was developed by Wu et al. [39] in 2007. Further well-designed, randomized, double-blind, placebo-controlled trials need to be carried out and reported in detail according to CONSORT or CONSORT for TCM. Fourth, various kinds of different forms of CHMs were tested in the 19 studies included, without detailed information on composition, dosage preparation, and manufacturing standards, and so forth. Thus, it is necessary to identify which one of the herbs displays an anti-Parkinsonian action and find the active component of this herb medicine. In this way, we can assess the effect of a particular CHM by means of the evidence synthesis of trials.

## Figures and Tables

**Table 1:** Characteristics of the included studies.

			Interventions (*n*)		Samples and Characteristics		Hoehn and Yahr				
Included trials	Eligibility criteria1	Study Designs	Drug/dosage		Male/female (*n*); age (years); duration (years)		Staging scale (stage) : case (*n*)		Course of treatment	Outcomes	Intergroup differences
			Trial	Control	Trial	Control	Trial	Control			
Cui et al. [17] CN, ADJ monocenter	CNDS for PD in 1984	Randomized (stratified randomized) and controlled nonblind parallel study	BuShenPing ChanFang 1 dose/d^#^	Madopar 62.5–500 mg/per time (pt), bid-qid	M31/F21 Mean age: 67.9 ± 16.5 Disease duration: 2–12	M25/F10 Mean age: 65.5 ± 16.5 Duration: 1–10	1.5–3 : 22 4 : 13	1.5~3 : 25 4 : 10	3 mon	(1) UPDRS (2) Clinical symptom (3) Adverse effect*	(1) *P* < 0.05 (2) *P* < 0.05 or *P* < 0.01
Wang et al. [18] CN, ADJ monocenter	CNDS for PD in 1984	Randomized (simple randomized) and controlled parallel study	ZiYinXiFeng HuoXueTang 1 dose/d^#^	Madopar 125 mg/pt for 1 week; then 250 mg/ pt bid	M11/F9 Age range: 45–74 Disease duration: 7 mo–6 y	M10/F10 Age range: 42–75 Disease duration: 6 mo–5 y	nr	nr	3 mon	(1) UPDRS (2) Tremble function	(1) *P* < 0.01 (2) *P* < 0.01
Wang et al. [19] CN, ADJ monocenter	CNDS for PD in 1984	Randomized (method unreported) and controlled nonblind parallel study	CHM according to syndrome differentiation^#^	Madopar <3 piece/d; If stiff, add amantadine 0.1-0.2/d	M33/F20 Mean age: 63.91 ± 9.3 Disease duration: nr	M30/F20 Mean age: 62.62 ± 9.54 Disease duration: nr	nr	nr	90 d	(1) UPDRS II, III (2) Clinical symptom	(1) *P* < 0.05 (2) *P* < 0.05

Shen and Yuan [20] CN, ADJ monocenter	CNDS for PD in 1984	Randomized (method unreported) and controlled parallel study	ZiBuGanShen 1 dose/d^#^	Madopar, Sinemet, Artane; no detailed information concern on the dosage	M26/F14 Mean age: 71.30 ± 6.92 Disease duration: 4.30 ± 2.31	M20/F10 Age range: 67.91 ± 7.64 Disease duration: 3.91 ± 2.01	2 : 8 2.5 : 18 3 : 10 4 : 4	2 : 6 2.5 : 15 3 : 6 4 : 5	3 mon	(1) UPDRS II, III (2) Webster scale	(1) *P* < 0.05 (2) *P* < 0.05

Luo et al. [21] CN, ADJ monocenter	CNDS for PD in 1984	Randomized (simple randomized) and controlled nonblind parallel study	PaBing I Hao 1 dose/d^#^	Madopar; no detailed information concern on the dosage	M18/F4 Mean age: 64.54 ± 10.61 Disease duration: 5.19 ± 5.22	M12/F7 Mean age: 66.80 ± 9.15 Disease duration: 5.57 ± 3.56	1 : 6 2 : 10 3 : 3 4 : 3	1 : 4 2 : 10 3 : 3 4 : 2	3 mon	(1) UPDRS	(1) *P* < 0.05

Zheng and Luo [22] CN, ADJ monocenter	CNDS for PD in 1984	Randomized (simple randomized) and controlled nonblind parallel study	PaBing III Hao 1 dose/d^#^	Madopar 125 mg/pt, bid for 1 w; then 250 mg/pt, bid	M15/F15 Mean age: 63.43 ± 10.09 y Disease duration: 6 mo–15 y	M19/F11 Mean age: 62.30 ± 6.82 y Disease duration: 9 mo–13 y	1 : 12 2 : 11 3 : 7	1 : 6 2 : 15 3 : 9	3 mon	(1) UPDRS (2) Hoehn-yahr class	(1) *P* < 0.05 (2) *P* > 0.05

Xie et al. [23] CN, ADJ monocenter	CNDS for PD in 1984	Randomized (method unreported) and controlled nonblind parallel study	JunFuKangJiao Nang 1.5 g/per time (pt), tid #	Madopar 125 mg/pt, tid	M: 8/F: 6 Mean age: 59.00 ± 9.64 y Disease duration: nr	M: 7/F: 7 Mean age: 58.6 ± 12.0 y Disease duration: nr	nr	nr	6 mon	(1) UPDRS (2) Adverse effect (3) Clinical symptom	(1) *P* < 0.01 (2) nr (3) *P* < 0.05

Cheng et al. [24] CN, ADJ monocenter	CNDS for PD in 1984	Randomized (method unreported) and controlled nonblind parallel study	XiFengDingChan Wan 6 g/pt, tid #	Madopar for 12 w; convention dosage add the dosage when effect decline dosage subtract 62.5 mg/d over the 2 weeks	M12/F8 Mean age: 63 ± 6.07 Disease duration: nr	M11/F9 Mean age: 60.35 ± 6.73 Disease duration: nr	nr	nr	12 w	(1) UPDRS (2) Madopar dosage	(1) *P* < 0.01 (2) *P* < 0.01

Zhu et al. [25] CN, ADJ monocenter	CNDS for PD in 1984	Randomized (method unreported) and controlled nonblind parallel study	DingZhenTang 1 dose/d^#^	Antiparkinsonian drug, no detailed information	M23/F11 Mean age: 72.2 ± 6.7 Disease duration: 3.3 ± 2.4	M21/F10 Mean age: 70.0 ± 7.6 Disease duration: 3.5 ± 2.5	1 : 4 1.5 : 4 2 : 7 2.5 : 10 3 : 8	1 : 4 1.5 : 3 2 : 6 2.5 : 9 3 : 9	6 mo	(1) UPDRS (2) Autonomic nerve function	(1) *P* = 0.0018 (2) *P* > 0.05 (constipation *P* < 0.01)

Zhao et al. [26] CN, ADJ Multicenter	CNDS for PD in 1984	Randomized (case randomized) and controlled blind parallel study	GuiLingPaAnJiao Nang 3 pill/pt tid^#^	Placebo 3 pill/pt tid; Madopar, Sinemet plus Placebo 3 pill/pt tid	M46/F29 Mean age: 64.86 ± 9.85 Disease duration: 4.27 ± 3.44	M47/F32 Mean age: 65.63 ± 8.51 Disease duration: 4.59 ± 3.82	nr	nr	12 w	(1) UPDRS II, III, total (2) Levodopa dosage (3) Clinical effect	(1) *P* < 0.05 or *P* < 0.01 (2) *P* < 0.05 (3) *P* > 0.05

Kum et al. [27] HK, ADJ monocenter	UK Brain Bank standard	Randomized (computer- generated randomized) and controlled double-blind parallel study	JiaWeiLiuJunZi Tang; no detailed information concern on the dosage^#^	Placebo with each dose of their levodopa treatment	M14/F8 Mean age: 64.82 ± 8.88 y Disease duration: 5.44 ± 5.26	M17/F8 Mean age: 60.88 ± 9.41 Disease duration: 5.36 ± 5.27	nr	nr	24 w	(1) PDQ-39 (2) UPDRS (3) GDS (4) SF-36 (5) DSQS	(1) *P* < 0.05 (2) *P* < 0.05 (3) *P* > 0.05 (4) *P* > 0.05 (5) *P* > 0.05

Yang et al. [28] CN, ADJ Multicenter	UK Brain Bank standard	Randomized (central random System randomized) and controlled blind parallel study	BuShenHuoXue Particle 1 dose/d^#^	Madopar 375–1000 mg/pt tid-qid; placebo 1 dose/d	M29/F26 Mean age: 66.4 ± 9.1 Disease duration: 5.3 ± 3.1	M35/F16 Mean age: 67.5 ± 9.5 Disease duration: 5.0 ± 3.9	nr	nr	3 mon	(1) UPDRS III (2) Movement experiment (3) 10 m reentry run (4) Muscular tension	(1) *P* < 0.05 (2) *P* > 0.05 (3) *P* < 0.05 (4) *P* < 0.05

Yuan et al. [29] CN, ADJ Multicenter	CNDS for PD in 1984	Randomized (block randomization) and controlled blind parallel study	ShuDiPingChan Tang 2 bag/ptbid; XieWuJiaoNang 8 pill/pt, bid^#^	Antiparkinsonian drug; no definite information	M18/F12 Mean age: 69.5 ± 7.81 Disease duration: 7.43 ± 1.64	M16/F14 Mean age: 68.6 ± 7.32 Disease duration: 7.35 ± 1.82	2 : 10 2.5 : 28 3 : 12 4 : 10	2 : 12 2.5 : 30 3 : 8 4 : 10	3 mon	(1) UPDRS (2) Clinical symptom (3) H-Y Stage (4) On-off phenomenon (5) Levodopa dosage	(1) *P* < 0.01 (2) *P* < 0.01 (3) *P* < 0.05 (4) *P* < 0.01 (5) *P* > 0.05

Hong [30] CN, ADJ monocenter	CNDS for PD in 2006	Randomized (method unreported) and controlled nonblind parallel study	CHM, No detailed information^#^	Madopar 62.5–125 mg/pt, bid-tid; Adjust the dosage according to the effect	M23/F15 Mean age: 72.2 ± 6.6 Disease duration: 19.6 ± 5.4	M21/, F17 Mean age: 73.1 ± 6.9 Disease duration: 20.5 ± 5.8	nr	nr	6 mon	(1) UPDRS III (2) UPDRS IV	(1) *P* < 0.05 (2) *P* < 0.05

Fan et al. 2010 [31] CN, ADJ monocenter	CNDS for PD in 2006	Randomized simple randomized) and controlled nonblind parallel study	PaBing II Hao 1dose/d continue 3 w; interval 1 w for 3 mon^#^	Madopar 125 mg/pt, bid for 1 w; Then 250,125,125 mg/pt (morning, middle, night, resp.) for 3 mon	30 PD Mean age: nr Disease duration: nr	30 PD Mean age: nr Disease duration: nr	nr	nr	3 mon	(1) UPDRS (2) Clinical symptom	(1) *P* < 0.05 (2) *P* < 0.05

Dou and Diao [32] CN, ADJ monocenter	CNDS for PD in 1984	Randomized (method unreported) and controlled nonblind parallel study	BuShenHuoXue Tang 600 mL/d^#^	Madopar 125 mg/pt, tid	M22/F13 Mean age: 54.7 ± 11.5 Disease duration: 3.7 ± 1.9	M21/F14 Mean age: 53.5 ± 11.9 Disease duration: 3.4 ± 1.8	nr	nr	3 mon	(1) UPDRS (2) Clinical symptom	(1) *P* < 0.05 (2) *P* < 0.05

Li et al. [33] CN, ADJ monocenter	UK Brain Bank standard	Randomized (method unreported) and controlled nonblind parallel study	BuShenHuoXueYin 1 dose/d divide twice 150 mL/pt^#^	Madopar 125 mg–1500 mg/d; Sinemet 125 mg–500 mg/d; Benzhexol 2–6 mg/d; Amantadine100–300 mg/d; Trastal 50–150 mg/d;	M30/F17 Mean age: 65.2 ± 7.8 y Disease duration: 5.61 ± 4.18	M25/F19 Mean age: 65.3 ± 8.8 y Disease duration: 5.97 ± 4.24	1.5 : 4 2 : 16 2.5 : 14 3 : 10 4 : 3	1.5 : 3 2 : 16 2.5 : 15 3 : 8 4 : 2	3 mon	(1) UPDRS total (2) UPDRS II (3) UPDRS III (4) Clinical effect (5) H-Y stage (6) Motor complications	(1) *P* < 0.05 (2) *P* < 0.05 (3) *P* > 0.05 (4) *P* < 0.05 (5)*P* > 0.05 (6) Reduced

Wu et al. [34] CN, ADJ monocenter	CNDS for PD in 2006	Randomized (random- number table) and controlled nonblind parallel study	ZhichanpingPaTang 1 dose/d, divide twice 600 mL/d^#^	Madopar 125 mg/pt, bid for 1 w; then 250, 125, 125 mg/pt (morning, middle, night, resp.)	M20/F20 Mean age: 69.28 ± 10.21 Disease duration: 3.94 ± 3.02	M22/F18 Mean age: 68.8 ± 7.59 Disease duration: 4.44 ± 3.17	1–2.5 : 29 3 : 11 4-5 : 0	1–2.5 : 28 3 : 12 4-5 : 0	3 mon	(1) UPDRS I (2) UPDRS II (3) UPDRS III (4) UPDRS IV (5) LSIB scale (6) PDQ	(1) *P* < 0.05 (2) *P* < 0.05 (3) *P* < 0.05 (4) *P* < 0.05 (5) *P* < 0.05 (6) *P* < 0.05

Pan et al. [35] CN, ADJ monocenter	UK Brain Bank Standard	Randomized (random numbers) and controlled blind parallel study	Zengxiao Anshen Zhichan 8 g/d^#^	Placebo granule; antiparkinsonian drug	M34/F22 Mean age: 62.82 ± 10.31 Disease duration: 5.73 ± 4.81	M21/F14 Mean age: 63.1 ± 10.2 Disease duration: 5.81 ± 3.24	nr	nr	13 w	(1) AMI counts (2) UPDRS II, III, IV (3) Power-law Exponent *α* (4) Secondary symptom score	(1) *P* < 0.05 (2) *P* < 0.05 (3) *P* < 0.01 (4) *P* < 0.05 or *P* < 0.01

CN: China, ADJ: adjunctive; CNDS: Chinese National Diagnosis Standard; PD: Parkinson's disease; RCT: randomized controlled trial; nr:  no report; w:  weeks; mon:  months; UPDRS:  Unified Parkinson's Disease Rating Scale; M:  male; F:  female; PDQ-39: Parkinson's Disease Questionnaire-39; GDS: Geriatric Depression Scale; SF-36: Short-Form-36 Health Survey; DSQS: Deficiency of Splenic Qi Scale; TCM:  traditional Chinese medicine; CHM:  Chinese herbal medicine; H-Y stage: Hoehn and Yahr stage; AMI: Ambulatory Monitoring Inc. ^#^: mean same as the control group treatment; *: adverse effect showed in Table 4.

**Table 2:** The included trials scored according to the risk of bias criteria.

	A	B	C	D	E	F	G	H	I	J	K	L	Total +/12	Total −/12	Total ?/12
Cui et al. [17]	+	−	−	−	−	?	?	?	+	+	?	+	4	4	4
Wang et al. [18]	+	+	−	−	−	?	?	?	?	?	+	+	4	3	5
Wang et al. [19]	?	−	−	−	−	?	?	?	?	+	?	+	2	4	6
Shen and Yuan [20]	?	−	−	−	−	?	?	?	+	+	?	+	3	4	5
Luo et al. [21]	?	−	−	−	−	?	?	?	+	+	+	+	4	4	4
Zheng and Luo [22]	?	−	−	−	−	?	?	?	+	+	+	+	4	4	4
Xie et al. [23]	?	−	−	−	−	?	?	?	?	+	+	+	3	4	5
Cheng et al. [24]	?	−	−	−	−	?	?	?	?	+	?	+	2	4	6
Zhu et al. [25]	?	−	−	−	−	?	?	?	+	?	?	+	2	4	6
Zhao et al. [26]	+	+	+	+	+	+	?	+	+	+	+	+	11	0	1
Kum et al. [27]	+	−	+	+	−	+	+	?	+	+	+	+	9	2	1
Yang et al. [28]	+	+	+	+	−	+	+	?	+	+	+	+	10	1	1
Yuan et al. [29]	+	+	+	+	−	?	?	?	+	?	+	+	7	1	4
Hong [30]	?	−	−	−	−	?	?	?	?	+	?	+	2	4	6
Fan et al. [31]	−	−	−	−	−	?	?	?	?	+	+	+	3	5	4
Dou and Diao [32]	?	−	−	−	−	?	?	?	?	+	+	+	3	4	4
Li et al. [33]	?	−	−	−	−	?	?	?	+	+	+	+	4	4	4
Wu et al. [34]	+	−	−	−	−	?	?	?	+	+	+	+	5	4	3
Pan et al. [35]	+	+	+	+	−	?	+	?	+	?	?	+	8	0	4

A: adequate sequence generation; B: concealment of allocation; C: blinding (patient); D: blinding (investigator); E: blinding (assessor); F: incomplete outcome data addressed (ITT analysis); G: incomplete outcome data addressed (dropouts); H: free of selective reporting; I: similarity at baseline; J: cointerventions constant; K: compliance acceptable; L: similar timing outcome assessments. +: yes, −: no, ?: unclear.

**Table 3:** The 16 herbs used more than 3 times for PD in the 19 trials included.

Chinese Pinyin	Latin herb name	English herb name	Frequency	The total frequency (127)%	Dosage
Dihuang	Radix Rehmanniae preparata	Prepared Rehmannia Root	10	7.9	10–24 g
Baishao	Radix Paeoniae Alba	White peony Alba	10	7.9	12–30 g
Chuanxiong	Rhizoma Chuanxiong	Szechwan Lovage Rhizome	10	7.9	12–15 g
Tianma	Rhizoma Gastrodiae	Tall Gastrodis Tuber	9	7.1	10–20 g
Gouteng	Ramulus Uncariae Cum Uncis	Gambir Plant	6	4.7	15–20 g
Danggui	Radix Angelicae Sinensis	Chinese Angelica	6	4.7	10–20 g
Heshouwu	Radix Polygoni Multiflori	Fleeceflower Root	5	3.9	15–20 g
Shanzhuyu	Fructus Corni	Asiatic Cornelian Cherry Fruit	5	3.9	8–20 g
Shichangpu	Rhizoma Acori Tatarinowii	Grassleaf Sweetflag Rhizome	4	3.1	10 g
Quanxie	Scorpio	Scorpion	4	3.1	1.5–10 g
Jiangcan	Bombyx Batryticatus	Stiff Silkorm	4	3.1	9–15 g
Danshen	Radix Salviae Miltiorrhizae	Danshen Root	4	3.1	10–15 g
Wumei	Fructus Mume	Smoked Plum	4	3.1	9–15 g
Huanglian	Rhizoma Coptidis	Golden Thread	3	2.4	9–15 g
Roucongrong	Herba Cistanches	Desertliving Cistanche	3	2.4	10–15 g
Tiannanxing	Rhizoma Arisaematis	Jackinthepulpit Tuber	3	2.4	10–15 g

**Table 4:** Forest plot of comparison: Chinese herbal medicine versus conventional treatment: UPDRS I scores.

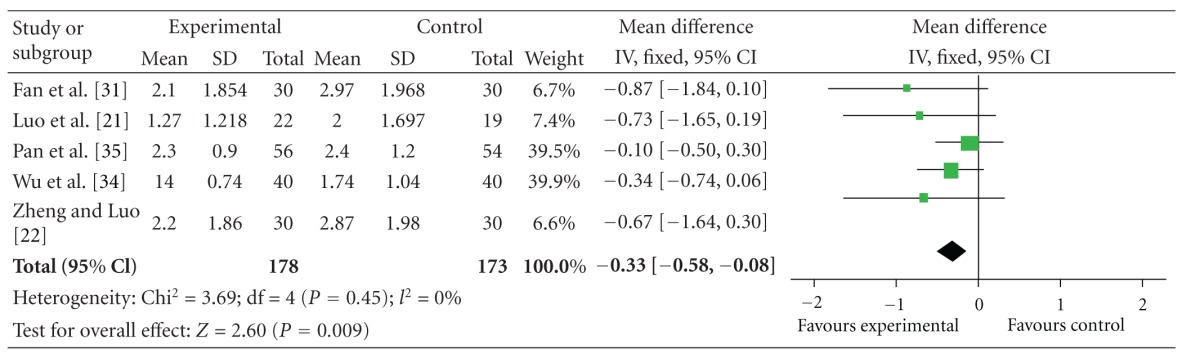

**Table 5:** Forest plot of comparison: Chinese herbal medicine versus conventional treatment: UPDRS II scores.

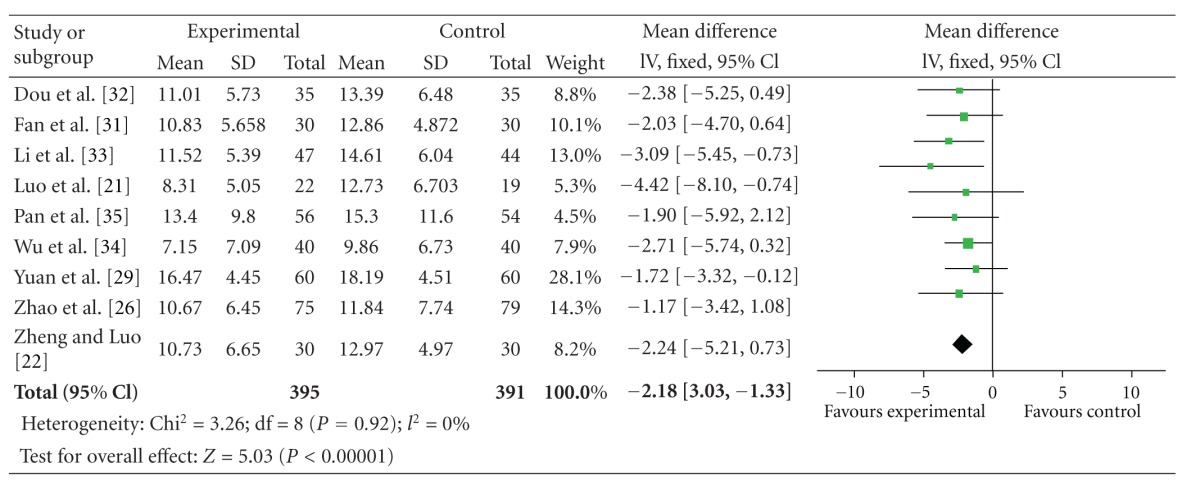

**Table 6:** Forest plot of comparison: Chinese herbal medicine versus conventional treatment: UPDRS III scores.

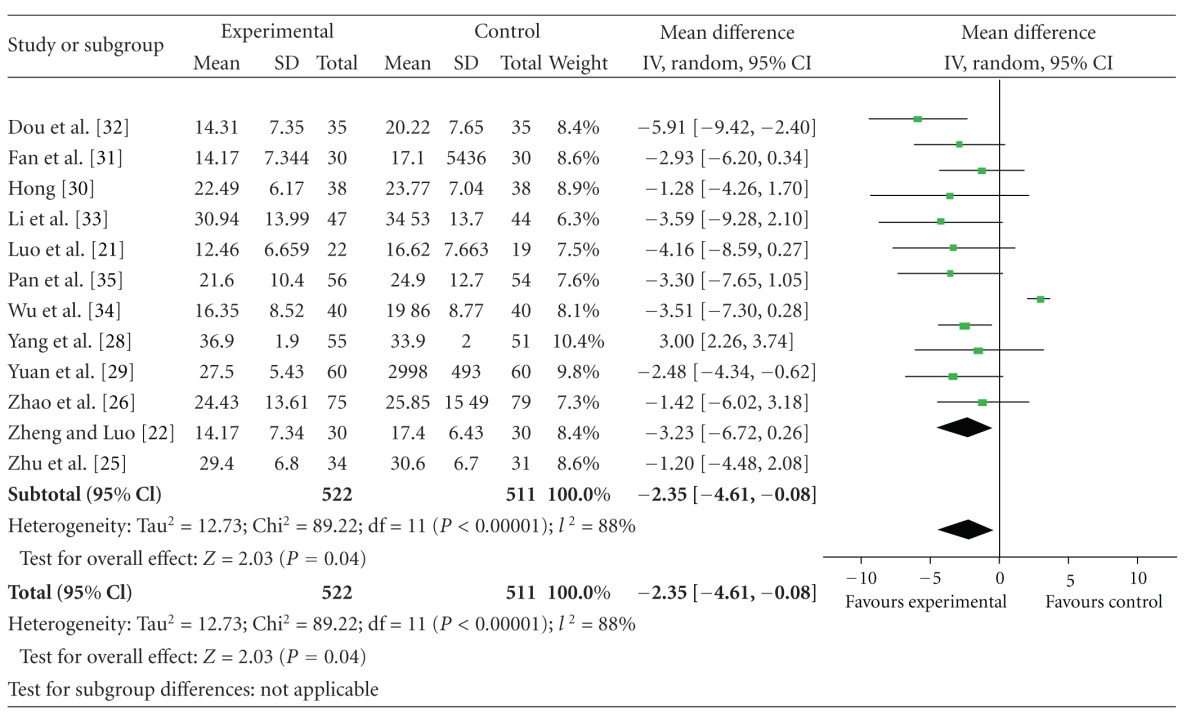

**Table 7:** Forest plot of comparison: Chinese herbal medicine versus conventional treatment: UPDRS IV scores.

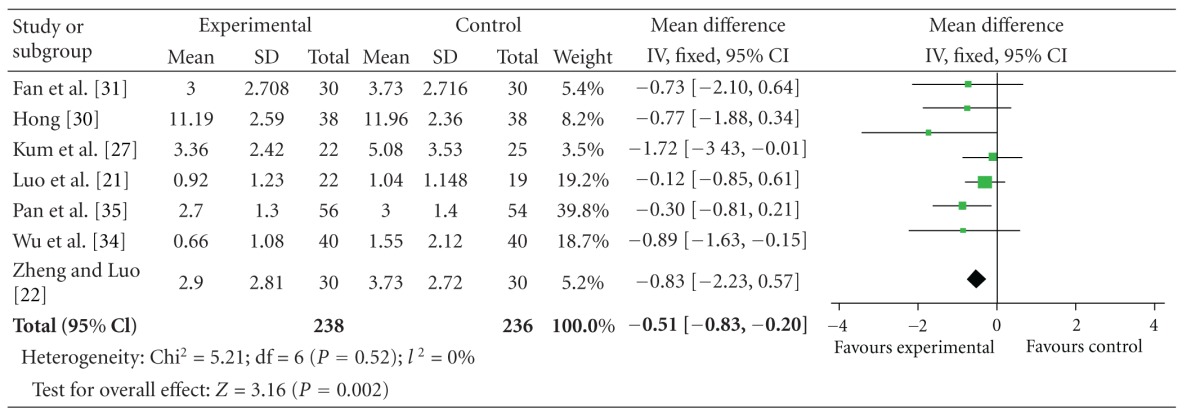

**Table 8:** Forest plot of comparison: Chinese herbal medicine versus conventional treatment: UPDRS I–IV total scores.

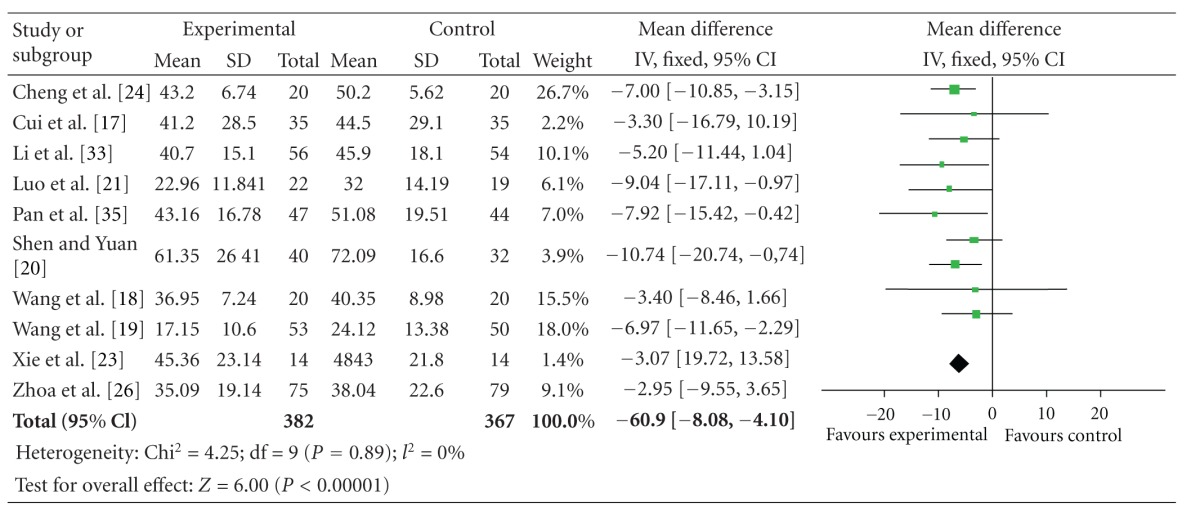

**Table 9:** Adverse effects found in CHMs for PD in the 19 trials included.

	Adverse drugs reaction	
	Trial	Control
Cui et al. [17]	Slight dry mouth, nausea, dizziness, tolerable, 2 cases. No significant change in BP before and after treatment (*P* > 0.05)	Nausea, spontaneous remission, 5 cases. Mild dizziness, spontaneous remission, 2 cases. No significant change in BP before and after treatment (*P* > 0.05)

Wang et al. [18]	No report	No report

Wang et al. [19]	No report	No report

Shen and yuan [20]	Nausea and vomiting, 5 cases (12.5%). Constipation, 8 cases (20%). Dry mouth, 4 cases (10%). No significant difference in blood and urine routine, liver and kidney function, and ECG before and after treatment (*P* > 0.05)	Nausea and vomiting, 11 cases (34.4%). Constipation, 13 cases (40.6%). Dry mouth, 5 cases (15.6%). No significant difference in blood and urine routine, liver and kidney function, and ECG before and after treatment (*P* > 0.05)

Luo et al. [21]	No report	No report

Zheng and Luo [22]	1/3 patients of both two groups received examinations such as blood routine, urine routine, electrocardiogram,	
	and liver and kidney function tests. No abnormal changes directly related to the treatment were found.	

Xie et al. [23]	The onset of symptoms such as nausea, vomiting, dizziness, headache, constipation, psychiatric symptoms, and	
	on-off phenomenon is less in treatment group than in control group.	

Cheng et al. [24]	Slight nausea, arrhythmia and dizziness, 2 cases. Spontaneous remission after two weeks	Nausea, constipation, 6 cases. Mild dizziness and arrhythmia, 3 cases. Spontaneous remission

Zhu et al. [25]	No report	No report

Zhao et al. [26]	No report	No report

Wan et al. [27]	Most patients tolerated the study drug well. One patient in the TCM group suffered from mild diarrhea. No other	
	adverse effects were reported by patients	

Yang et al. [28]	No significant changes in blood routine, urine routine, liver and kidney function and ECG before and after treatment. Mild diarrhea, 2 cases. Spontaneous remission after one day	No significant changes in blood routine, urine routine, liver and kidney function and ECG before and after treatment. Adverse reactions in 6 cases (not described in detail)

Yuan et al. [29]	Gastrointestinal side effects such as mild nausea or upper abdominal pain, 14 cases (*P* > 0.05). Mild and tolerable. No withdrawal due to adverse events. Constipation, 22 cases, relived after orally taking Maren pills or Glycerine enema. No significant changes in HR, BP, ECG, and liver and kidney function before and after treatment	Gastrointestinal side effects such as mild nausea or upper abdominal pain, 10 cases (*P* > 0.05). Mild and tolerable. No withdrawal due to adverse events. Constipation, 25 cases, relived after orally taking Maren pills or Glycerine enema. No significant changes in HR, BP, ECG, and liver and kidney function before and after treatment

Hong [30]	No report	No report

Fan et al. [31]	No report	No report

Dou and Diao [32]	Nausea, 3 cases. Salivation, 3 cases. Hypotension, 1 case. Insomnia, 4 cases. Depression, 3 cases	Nausea, 5 cases. Salivation, 5 cases. Hypotension, 6 cases. Insomnia, 12 cases. Depression, 5 cases. On-off phenomenon, 1 case

Li et al. [33]	No report	No report

Wu et al. [34]	No report	No report

Pan et al. [35]	Neither physical examination nor laboratory tests revealed any adverse changes after additional treatment in either	
	group	

BP: blood pressure; ECG: electrocardiography; HR: heart rate.
